# Exploratory clinical trial to evaluate the efficacy and safety of carbon dioxide paste in healthy people

**DOI:** 10.1097/MD.0000000000029511

**Published:** 2022-07-22

**Authors:** Nanae Yatagai, Takumi Hasegawa, Katsusuke Kyotani, Tomohiro Noda, Rika Amano, Izumi Saito, Satomi Arimoto, Daisuke Takeda, Yasumasa Kakei, Masaya Akashi

**Affiliations:** a Department of Oral and Maxillofacial Surgery, Kobe University Graduate School of Medicine, Kobe, Hyogo, Japan; b Center for Radiology and Radiation Oncology, Kobe University Hospital, Kobe, Japan.

**Keywords:** blood flow, clinical trial, hypoxia, postoperative pain, transcutaneous CO_2_

## Abstract

Scarring and pain are postoperative complications in patients after head and neck cancer treatment; however, there is no effective treatment. These complications are affected by local blood flow disorders, and it is well known that the transcutaneous application of carbon dioxide (CO_2_) improves local blood flow. Previously, we have shown that the transcutaneous application of carbon dioxide causes absorption of CO_2_ and increase the oxygen (O_2_) pressure in the treated tissue; it is expected that the application of CO_2_ may reduce scarring and pain caused by cancer treatment. We newly introduced the CO_2_ paste as a new CO_2_ application method, which does not need to use CO_2_ gas directly. In this study, we aimed to apply of CO_2_ paste to healthy people and to investigate its usefulness, safety and feasibility by analysing the increase in blood flow and frequency of adverse events.

**Methods:**

We applied carbon dioxide paste to skin over the sternocleidomastoid and gastrocnemius muscles of eight healthy volunteers. The changes in blood flow before and after the CO_2_ paste application using dynamic MRI, and changes in the vital signs were evaluated.

**Results:**

In the neck area and middle layer of the lower leg, the signal intensity (SI) significantly increased 60 seconds after application. In the surface layer of the lower leg, the SI was significantly increased 60 and 300 seconds after paste application. Although mild heat was noted after the paste application, no obvious adverse events occurred.

**Conclusion:**

We demonstrated the increase in SI by dynamic MRI at the site of the carbon dioxide paste application, which indicates the paste application is effective in improving the blood flow.

## 1. Introduction

The current main treatments for head and neck cancer are surgery, chemotherapy and radiotherapy. Postoperative complications, such as scarring and pain, which remain for a long time after these treatments, cause physical and psychological suffering for patients, even though the cancers are completely cured. Chronic pain, such as postoperative pain, is associated with vascular dysfunction, and there are many reports about microvascular disturbances in neuropathic pain animal models and humans with neuropathic pain.^[1–3]^ In rats, the restriction of blood flow and tissue ischemia cause complex regional pain syndrome-like symptoms.^[1]^ In humans, people with sickle cell anaemia experience extreme pain during vaso-occlusive episodes, whereby sickle-shaped red blood cells obstruct blood vessels, resulting in tissue ischemia.^[2]^ Chronic ischemic pain is also reported in patients with peripheral arterial diseases.^[3]^ Chronic pain after neck dissection is considered to be related to local vascular disturbances other than surgical neuropathy. Various medications have been used to improve these postoperative neuropathic pains^[4–7]^; however, they have some side effects and they insufficiently reduce chronic pain.^[8,9]^ Therefore, none dramatically relieve postoperative suffering of cancer patients. Chronic postoperative pain may be resolved if the blood flow of scar tissue can be improved.

Previously, we reported the effects of the transcutaneous application of CO_2_ in a hydrogel as an absorption-promoting agent for healthy people and various animal models.^[10–12]^ The transcutaneous CO_2_ application increased the number of mitochondria, increased muscle fibre switching, promoted vascularization, and improved the exercise performance in rats.^[13]^ In other studies on healthy people, the CO_2_ application improved the microcirculation and increased the transcutaneous oxygen causing an artificial Bohr effect. CO_2_ causes dissociation of oxygen from haemoglobin, promoting local oxygenation.^[14]^ However, it is difficult to apply this method to the head and neck area because it requires covering the application site and using CO_2_ gas. Focusing on the effect of the transcutaneous CO_2_ application as described above, we introduced a CO_2_ paste for a CO_2_ effect in the head and neck area. This new application mechanism does not directly use CO_2_ gas, but rather uses a paste that reacts with the water on the skin surface, which then generates CO_2_ gas. In this study, we aimed to apply of CO_2_ paste to healthy people and to investigate its usefulness, safety and feasibility by analysing the increase in blood flow and frequency of adverse events.

## 2. Methods

### 2.1. Subjects

Eleven healthy volunteers participated in this study. The subjects were 9 males and 2 females, aged 24 to 35 years (mean, 28.1 ± 3.7 years). Subjects who met the following conditions were excluded; firstly, those allergic to drugs, secondly, those with peripheral artery disease, ischemia (Fontaine III-IV), thirdly, those with heart failure (NYHA class IV), fourthly, those who are pregnant or have the possibility to be pregnant, fifthly, those with severe respiratory disease, sixthly, those who have difficulty applying the paste, such as to large skin ulcers or those with significant skin diseases, seventhly, those with claustrophobia, and finally, those who were deemed inappropriate by the researcher or the attending physician. Each subject provided written informed consent before participating in this study. This study was approved and permitted by the Ethical Committee of Kobe University Graduate School of Medicine. The approval number is No.300013.

### 2.2. Study protocol

This study is a single-arm clinical trial. After informed consent, the study period was for a total of 5 weeks, including a pre-observation period within 4 weeks, application of the CO_2_ paste for 20 minutes, and a post-observation period for 1 week. First, the screening of the subjects was performed. After the screening, we applied CO_2_ paste to the subject's skin. The CO_2_ paste was applied to the neck area and the lower legs to examine the effect to deep muscles. CO_2_ paste was applied only once with fingers to the skin of the lower leg and neck surface, the areas corresponding to the sternocleidomastoid and gastrocnemius muscle. Twenty minutes after the application, the paste was removed by wiping or rinsing. We then evaluated the effect of the CO_2_ paste by dynamic MRI. Subjective symptoms, such as pain, heat sensation, and discomfort, were evaluated by a Visual analogue scale (VAS). The patient's vital signs were also checked before, 10 minutes after and 1 week after the application of the paste, including the blood pressure and oxygen saturation (SpO_2_).

### 2.3. CO_2_ application

In this study, we used CO_2_ paste invented by NeoChemir Inc. (“direCO_2_t”). The paste contains sodium bicarbonate and malic acid as components for generating CO_2_ gas. When we apply it to skin, the paste reacts with the water of the skin surface, generating CO_2_ gas. This CO_2_ paste was applied evenly on the skin of the neck and lower leg. Twenty minutes later, the pate was wiped off.

### 2.4. Dynamic MRI

All MR imaging was performed with a 3.0-tesla system (Achieva, Philips Medical Systems, Best, The Netherlands) using a 32-channel cardiac coil. The BOLD MR imaging protocol was a single-shot multi-echo gradient-echo echo-planar imaging sequence with fat suppression with the following parameters: repetition time (TR), 1000 ms; echo time (TE), 4 echoes at 6.9, 19.5, and 44.5 ms; slice thickness, 4 mm; field of vision (FOV), 200 × 400 mm; and imaging matrix, 115 × 128. The slice position was set at the proximal quarter of the lower left leg and the neck area was taken at the centre of the sternocleidomastoid muscle. We performed dynamic measurement with a temporal resolution of one measurement for a consecutive series for 700 measurements of the whole phase. The paste was applied in the MR gantry with the first 30 seconds as a baseline. Imaging was performed for about 10 minutes after paste application.

### 2.5. Image analysis

**Figure 1.**
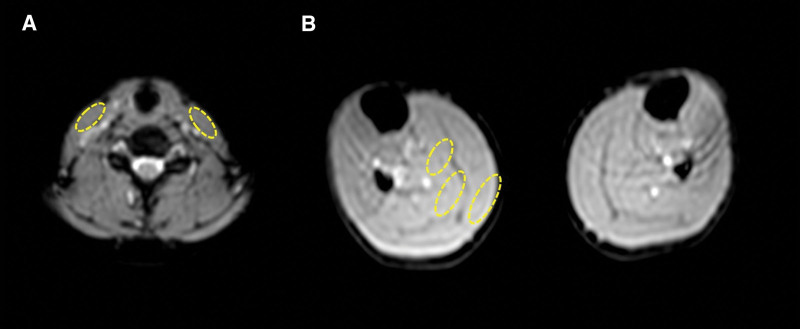
Regions of interest (ROI) in the MR images. (A) The ROI was set in the centre of sternocleidomastoid muscle in the neck area. (B) The ROI was set in three layers, superficial, middle and deep areas of gastrocnemius muscle in the lower leg.

We set regions of interest (ROI) in the MR images. In the neck area, the ROI was set at the centre of the sternocleidomastoid muscle. In the lower leg, the ROI was set in the three layers, superficial, middle and deep areas of the gastrocnemius muscle (Fig. 1). The data in the deep layer of the lower leg had large variations and errors, likely because the data were acquired near a large blood vessel. The reliability of the data in the deep layer was therefore uncertain and the data were excluded from the evaluation. The time points of the measurements were set to before paste application and 60, 300, and 700 seconds after paste application. We obtained the average SI in the ROI of 10 dynamics at each time point. Even before the application, there was variability in SI between the applicant side and the non-application side in the same region. Therefore, the SI before application in the paste application area was used as a control, and the relative value of the SI at each time point was determined according to the following steps. We defined this relative SI score as the signal intensity ratio. 1) Take the ROI on the right and left side of 1–10, 54–65, 294–305, 594–605 dynamics, respectively. 2) Calculate the average value of the right side ROI (1–10 dynamics)/left side ROI (1–10 dynamics). 3) Like Step 2, calculate the right side ROI/left side ROI of 54–65, 294–305, 594–605 dynamics. 4) Divide the value obtained in Step 3 by the value from Step 2. 5) Determine the median and standard deviation of the values obtained in Step 4. We evaluated the change in blood flow by the effect of CO_2_ paste using the signal intensity ratio.

### 2.6. Statistical analysis

Statistical analyses were performed with BellCurve for Excel (Social Survey Research Information Co., Ltd.). The results of the signal intensity ratio were analysed using the Mann–Whitney *U* test. Subjective symptoms and vital signs were analysed by Wilcoxon signed-rank test. The level of statistical significance was set at *P* < .05.

## 3. Results

**Figure 2.**
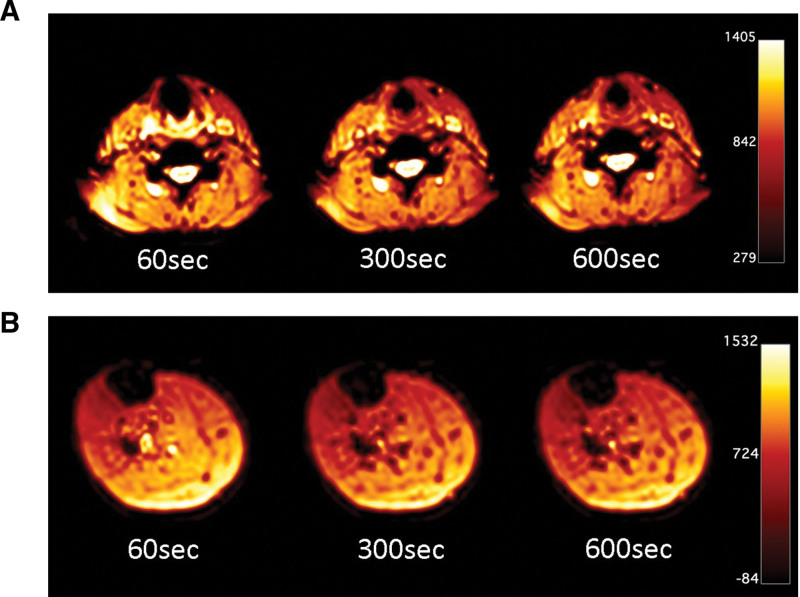
Proton density weighted images after carbon dioxide paste application. (A) Proton density weighted images in the neck area. (B) Proton density weighted images in the lower leg area.

Of 11 subjects, three were excluded due to body movement during MRI imaging that caused inaccurate imaging results. We analysed only data from 8 volunteers (Table 1). Dynamic MR imaging data showed improved blood flow at the paste application site on the neck and lower leg (Fig. 2). An improvement in the blood flow at the paste application site was observed over time. Blood flow was most improved in both the neck and gastrocnemius muscles 60 seconds after the paste was applied. Although a decrease in blood flow change over time was observed, blood flow improvement was still observed after 300 seconds and 600 seconds.

**Table 1:** Data of the subjects.

Volunteer	Sex	Complications	Age	Height (cm)	Weight (kg)
1	M	None	25	180	70
2	M	None	26	171	58
3	M	None	26	173	69
4	M	None	25	170	66
5	M	None	29	168	53
6	M	None	29	170	63
7	F	None	35	151	48
8	F	None	30	160	47
Mean			28.13	167.88	59.25
SD			3.40	8.77	9.16

F = female, M = male, SD = standard deviation.

### 3.1. Signal intensity ratio

**Figure 3.**
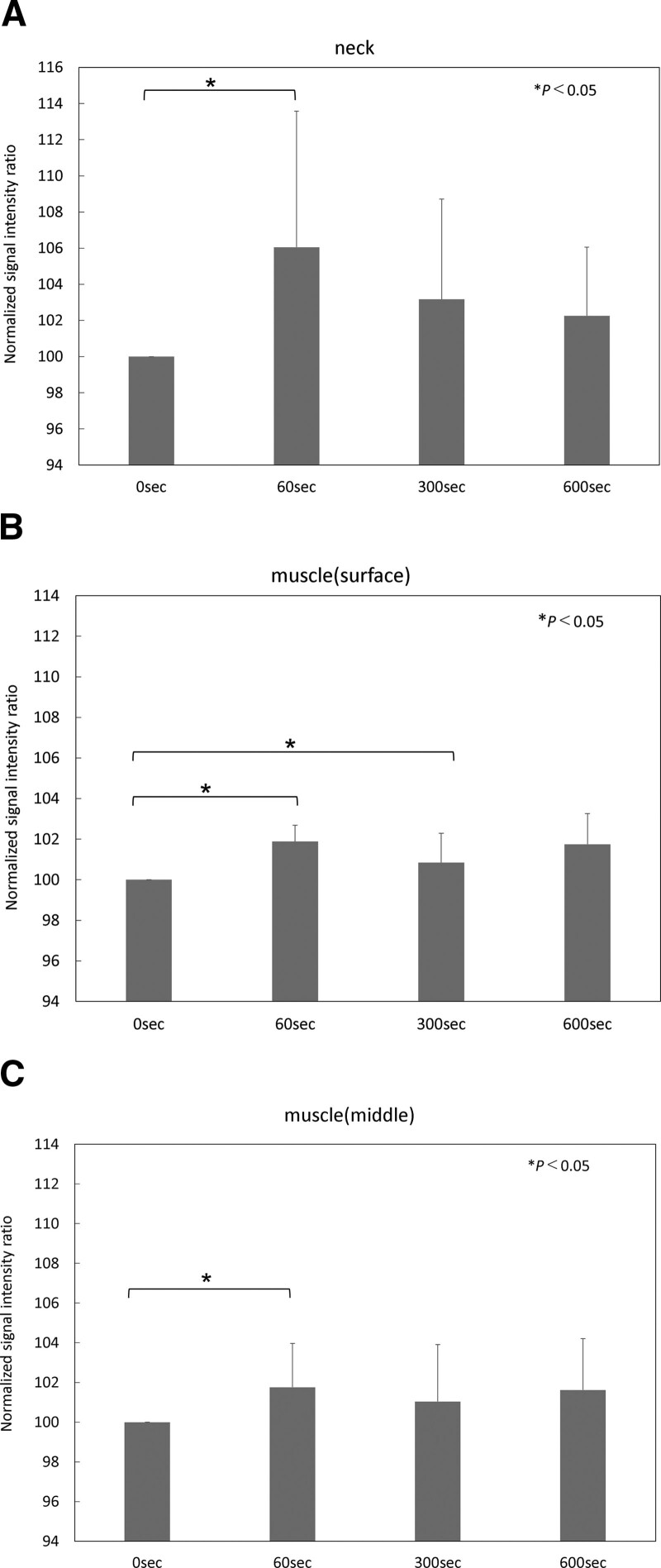
Normalized signal intensity ratio. (A) The graph of the normalized signal intensity ratio in the neck area. (B) The graph of the normalized signal intensity ratio in the surface layer of lower leg. (C) The graph of the normalized signal intensity ratio in the middle layer of lower leg.

The results of the signal intensity ratio of the eight subjects are shown in the following table. The average signal intensity ratio of the neck area at 60, 300, and 600 seconds after paste application are 106.1 ± 7.5%, 103.2 ± 5.5%, and 102.3 ± 3.8%, respectively. The average signal intensity ratio of the surface layer of the lower legs at each point are 101.9 ± 0.8%, 100.8 ± 1.5%, and 101.7 ± 1.5%. Those of the middle layer of the lower legs are 101.8 ± 2.2%, 101.0 ± 2.9%, and 101.6 ± 2.6%. In the neck and middle layer of the lower leg, a significant increase was observed in the signal intensity ratio 60 seconds after the paste application. In the surface layer of the lower leg, significant differences were observed 60 and 300 seconds after the application compared with the ratio before the application (Fig. 3).

### 3.2. Vital signs

**Figure 4.**
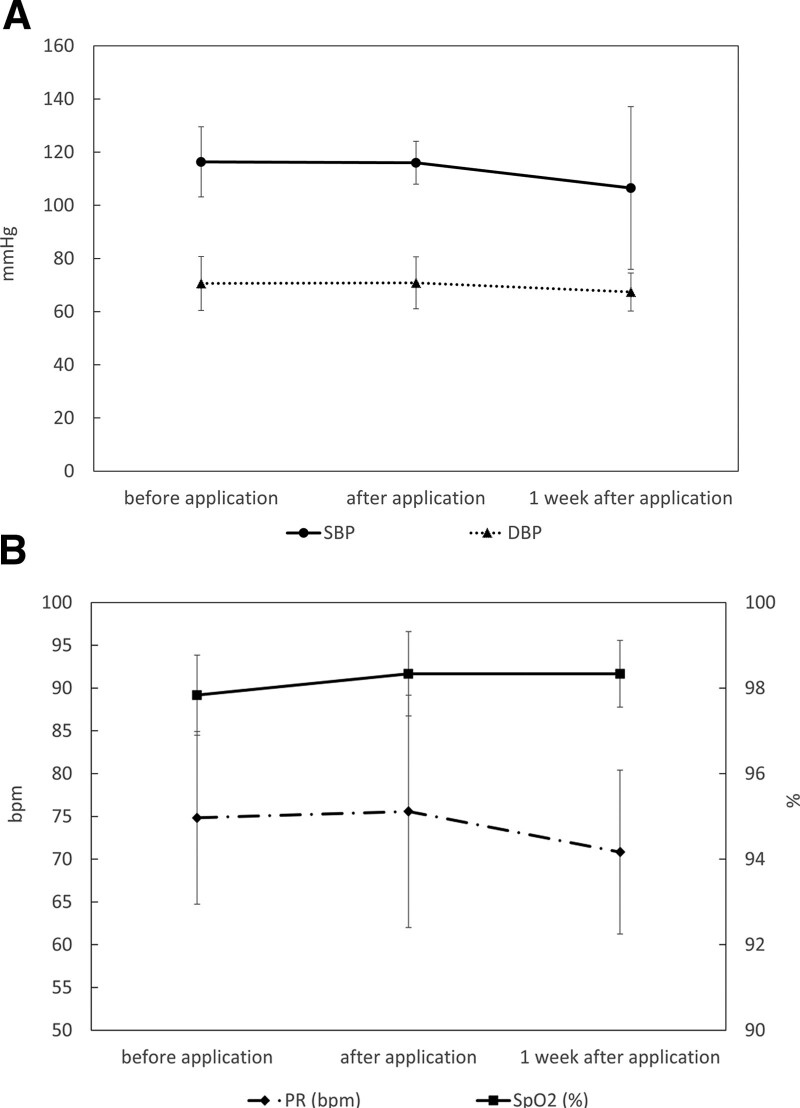
Average score of the vital signs (SBP, DBP and PR). (A) The graph shows the average SBP and DBP score of the 8 volunteers at each time point. (B) The graph shows the average PR and SpO_2_ score of the 8 volunteers at each time point. DBP = diastolic blood pressure, PR = pulse rate, SBP = systolic blood pressure.

There was no significant change in the pulse rate, blood pressure or SpO_2_ before, after and one week after application of the CO_2_ paste (Fig. 4).

### 3.3. Visual analogue scale (VAS)

Seven subjects recognized heat sensation at the paste application site in the neck and eight subjects recognized heat in the lower leg. The average VAS value in the subjects who felt the heat sensation was 1.86/10 (max: 5.42, min: .34). There were no complaints of pain or discomfort in all eight subjects (Tables 2 and 3).

**Table 2:** VAS in the neck area after carbon dioxide paste application.

	VAS		
Volunteer	Pain	Warmth	Discomfort
1	0	1.60	0
2	0	3.33	0
3	0	2.64	0
4	0	0.35	0
5	0	0	0
6	0	0	0
7	0	5.42	0
8	0	0	0
Mean	0	1.67	0
SD	0	1.99	0

SD = standard deviation, VAS = visual analogue scale.

**Table 3:** VAS in the lower leg area after carbon dioxide paste application.

	VAS		
Volunteer	Pain	Warmth	Discomfort
1	0	5.07	0
2	0	1.11	0
3	0	5.14	0
4	0	2.50	0
5	0	0	0
6	0	2.15	0
7	0	5.42	0
8	0	0	0
Mean	0	2.68	0
SD	0	2.28	0

SD = standard deviation, VAS = visual analogue scale.

## 4. Discussion

In this study, blood flow was increased by applying CO_2_ paste, which can transfer carbon dioxide transcutaneously into tissue without using pure CO_2_ gas. Although a mild sensation of heat occurred, no other side effects were observed.

Scars and pain caused by additional postoperative treatment are the most frequent side effects observed in head and neck cancer treatment. However, no effective treatment has been established. In this study, we investigated the effect of improving blood flow in the neck and lower legs using a new CO_2_ application system to improve hypoxia related to postoperative scar and pain.

Generally, pain that lasts long after surgery is called post-operative chronic pain and is said to occur in about 10 to 50% of various operations.^[15–21]^ Chronic pain is associated with poor general health, disability and depression, which can lead to social withdrawal and further comorbidities.^[22]^ In addition, postoperative scars and wounds significantly affect functional impairment and psychological burden,^[23]^ which can decrease the patient's quality of life.^[24]^ In patients with head and neck cancer, scars and scarring pain on the face and neck often occur due to the side effects of surgery and chemoradiotherapy. Myofascial pain syndrome occurs frequently in patients after cervical dissection.^[25]^ Therefore, postoperative scars and pain remain as a sequela even if the underlying disease is completely cured, which causes patient suffering. Lim et al observed the presence of persistent endoneurial hypoxia in a mouse model of traumatic peripheral nerve injury, causing painful mononeuropathy.^[26]^ Hypoxia is due to microvascular dysfunction, intimal fibrosis, and increased metabolic demand within the injured nerve. Increased lactate levels were observed in the injured nerves, as well as increased oxygen consumption and extracellular acidification rates. Hypoxia causes a reduction in levels of the Na+/K+ ATPase ion transporter in both cultured primary dorsal root ganglion neurons and injured peripheral nerve. A reduction in Na+/K+ ATPase ion transporter levels likely leads to the hyperexcitability of injured nerves.^[26]^ In another report, Kim et al demonstrated that the incision of the plantar hindpaw, gastrocnemius muscle, and paraspinal region increased the tissue lactate concentration in the rat models for incision-induced pain behaviours. The increased lactate could potentially facilitate nociceptor activation by low pH and contribute to pain after surgery.^[27]^

Surgical dissection of the neck and post-radiotherapy peripheral nervous system damage induce neuropathic pain.^[28]^ Postoperative pain after neck dissection is mostly due to nerve damage and muscle disorders.^[29]^ These postoperative pains are also associated with microvascular dysfunction. Various medications, such as ketamine^[4,5]^ and gabapentin,^[6,7]^ have been used to improve postoperative neuropathic pain. However, there is not enough evidence of their effectiveness for chronic pain.^[8]^ Moreover, they have some side effects; for example, ketamine can cause excessive sedation and psychedelic effects.^[9]^ Therefore, there are few reports on postoperative scarring and chronic pain in head and neck cancer patients, and effective treatment for these problems has not been established. There have been many research reports on carbon dioxide transcutaneous absorption methods. In rats, aerobic muscle migration, increased muscle weight, increased mitochondrial mass, increased angiogenesis, and improved exercise performance are observed.^[13]^ Previous studies conducted on healthy people have shown increased blood flow and artificially induced Bohr effects, increased oxygen dissociation from red blood cells, and local oxygenation.^[14]^ Recovery of muscle fatigue after delayed onset muscle soreness^[30]^ and increased muscle strength under non-exercise by long-term application were also observed.^[31]^ Furthermore, in a study using patients, there were reported cases in which transcutaneous absorption of CO_2_ gas resulted in improvements for fracture healing. Using a rat with a flap treatment on the back, Saito et al confirmed that the number of new blood vessels at the flap site and VEGF gene expression increase by using the carbon dioxide transcutaneous absorption method.^[12]^ Thus, the carbon dioxide transcutaneous absorption may prevent flap necrosis in connection with promoting angiogenesis and blood flow. As previously mentioned, the CO_2_ therapies are simple and safe as compared with past systemic medications and treatments. However, conventional methods require using carbon dioxide gas and were not applicable to the head and neck region. To investigate whether it could be a treatment for scarring and pain after head and neck cancer treatment, we examined the effect of CO_2_ paste treatment that can be applied to the head and neck area of healthy people. First, the paste was evaluated using dynamic MRI whether the blood flow improvement effect is the same as the conventional method using a carbon dioxide gas absorption gel, producing blood flow improvement. The effects of improving the blood flow in the neck region decreased sooner than that in the lower leg region. There are a lot of large blood vessels in the vicinity of the application site in the neck, which could influence the change in blood flow as the carbon dioxide paste application tends to disappear. In the lower leg, the influence of the blood flow change was greater in the surface layer than in the middle layer. The penetration of the paste is superficial, and it may not be possible to impact deep tissue. Although we performed the evaluation for only 600 seconds, it is necessary to further extend the duration of the effect of the hypoxic environment by CO_2_ paste for clinical application. As confirmed by the conventional method in animal experiments, whether the blood flow improves constantly by applying the CO_2_ paste repeatedly should be examined. Furthermore, the average age of patients with oral cancer is older than the age of the subjects (healthy people) in this study. Therefore, it is necessary to examine the effects of CO_2_ on actual patients with oral cancer because the effect of CO_2_ may differ from the results of this study. The CO_2_ paste used in this study is very safe, simple to use, and has been shown to be useful as a new CO_2_ transcutaneous absorption method applicable to the head and neck region. This paste is expected to be used clinically for the purpose of preventing skin flap necrosis, improving symptoms of wrinkles after radiotherapy and scarring after surgical treatment by utilizing its hypoxic environment improvement effect. This method may be applied to oral cancer patients, which may receive additional postoperative treatments such as radiotherapy and chemotherapy. It is also necessary to carefully study the influence of CO_2_ paste on tumours when combined with additional post-operative treatment.

## 5. Conclusions

In this study, an increase in blood flow was observed in dynamic MRI image evaluation by applying CO_2_ paste that can capture carbon dioxide percutaneously in tissue. No serious side effects were observed with the CO_2_ paste application, indicating its potential as a new treatment for scars and pain after head and neck treatment.

## Acknowledgments

We thank Ashleigh Cooper, PhD, from Edanz Group (https://en-author-services.edanzgroup.com/) for editing a draft of this manuscript.

## Author contributions

Conceptualization: Takumi Hasegawa

Data curation: Daisuke Takeda, Izumi Saito, Nanae Yatagai, Rika Amano, Satomi Arimoto, Yasumasa Kakei

Formal analysis: Katsusuke Kyotani, Tomohiro Noda

Funding acquisition: Takumi Hasegawa

Investigation: Daisuke Takeda, Izumi Saito, Nanae Yatagai, Rika Amano, Satomi Arimoto, Yasumasa Kakei

Methodology: Takumi Hasegawa

Project administration: Masaya Akashi, Takumi Hasegawa

Resources: Takumi Hasegawa

Supervision: Masaya Akashi

Validation: Katsusuke Kyotani, Tomohiro Noda

Visualization: Katsusuke Kyotani, Tomohiro Noda

Writing – original draft: Nanae Yatagai

Writing – review & editing: Takumi Hasegawa
